# Telemedicine and increased retention in medications for opioid use disorder treatment

**DOI:** 10.1186/s13722-026-00682-2

**Published:** 2026-06-17

**Authors:** Allison J. Smith, Sara M. Witcraft, Suzanne Lane, Constance Guille

**Affiliations:** Department of Psychiatry and Behavioral Sciences, Medical University of South Carolina, Charleston, SC USA

**Keywords:** Opioid use disorder, Telemedicine, Buprenorphine, Medications for opioid use disorder, Racial disparities

## Abstract

**Background:**

Buprenorphine is an effective treatment for Opioid Use Disorder (OUD), but many patients discontinue this medication within six-months of initiation and racial disparities in access and retention in buprenorphine treatment exist. Telemedicine is a potentially promising strategy to improve buprenorphine treatment retention.

**Objective:**

To determine if, compared to pre-pandemic in-person services, telemedicine visits were associated with increased retention in MOUD treatment at six-months and if there were differences by race.

**Methods:**

This is a retrospective cohort study that evaluated medical records of individuals treated for OUD in-person (*n* = 247) and via telemedicine (*n* = 204) at a large academic medical center in the Southeastern United States from 2018 to 2021. The primary outcome was the percentage of patients retained in treatment at six-months, defined as having an outpatient visit associated with a buprenorphine prescription. Chi-squared tests were used to evaluate the percentage of patients retained in treatment at six-months, multivariable logistic regression identified correlates of retention, and Kaplan-Meier survival analyses evaluated time in treatment.

**Results:**

35.3% of the cohort (*n* = 411; *M*_*age*_=38; 64% female; 89% White; 39% Medicaid insured) were retained in treatment within six-months of their index visit. Individuals with telemedicine-based treatment were 1.8 times more likely to be retained in treatment at six-months compared to those with in-person visits (AOR = 1.87, 95% CI = 1.17–3.00). Non-White individuals were 2.05 times more likely to remain in treatment at six-months, compared to White individuals (AOR = 2.05, 95% CI = 1.02–4.14).

**Conclusion:**

This study suggests that retention in treatment may be higher with telemedicine-based buprenorphine treatment compared to in-person care. Non-White patients appeared to experience an even greater benefit, though further research is needed to confirm this finding. As policymakers are determining telehealth prescribing laws, these findings further support the use of telemedicine to improve retention in effective treatment.

## Introduction

In the United States, opioid overdoses have claimed over 700,000 lives since 1999 [1]. Despite the availability of life-saving evidence-based treatments for Opioid Use Disorder (OUD), only a fraction (less than 20%) of those diagnosed with OUD in the past year will receive medications [2]. Significant racial, geographic, and socioeconomic disparities in receipt of this treatment exist with Black, low-income, and rural populations being less likely to receive MOUD [2–4]. Demographic profiles of those dying from drug overdoses demonstrate significant racial disparities predominantly affecting minoritized and under-represented groups. Between 2019 and 2020, overdose deaths increased by 44% and 39% among non-Hispanic Black and non-Hispanic American Indian/Alaskan Native (AI/AN) persons, respectively, compared to 22% among non-Hispanic White persons [2, 5]. Additionally, in South Carolina between 2014 and 2018, there was a *200-fold increase* in mortality burden for Black South Carolina decedents from opioid-involved overdose deaths compared to a 70-fold increase for White decedents [6]. 

Telemedicine is one strategy to expand access to medications for OUD (MOUD) (i.e., buprenorphine) for all patients, including those living in rural areas with limited healthcare providers, individuals with socioeconomic barriers to care, and non-White individuals who may be disproportionately impacted by stigma and discrimination [7]. However, the full potential of telemedicine for the treatment of OUD was not realized until the COVID-19 pandemic. During the pandemic, regulations for prescribing buprenorphine via telemedicine were temporarily removed to facilitate access to this life-saving treatment. In a large study of 175,778 Medicare recipients, the percentage of patients receiving OUD-telehealth related services was 35-fold that of a pre-pandemic cohort [8]. Recapitulating these findings, data from the 2021 National Survey on Drug Use and Health, representative of the United States population, found a nearly 38-fold increase in receipt of MOUD treatment delivered via telehealth compared to in-person [6]. Utilization of telehealth during the COVID-19 pandemic was associated with improved retention in OUD treatment and decreased overdoses, compared to a pre-pandemic cohort [8]. These large-scale studies suggest that use of telemedicine for the treatment of OUD expands access, improves retention in treatment, and reduces rates of overdose deaths. It is unclear, however, if telemedicine increases access and retention in treatment for all patients with OUD, especially among Black patients [7]. One study found the odds of retention in OUD telemedicine treatment for non-Hispanic Black individuals were lower compared to non-Hispanic White individuals for Medicaid enrollees in Kentucky and Ohio [9]. The same study also found that non-Hispanic Black individuals had lower adjusted odds of telemedicine initiation [9]. Another recent study showed that Black and Latinx enrollees in New York used fewer telehealth services for Substance Use Disorder (SUD) outpatient treatment compared to White patients [10]. 

There has been much discussion amongst policy makers on extension and adoption of tele-prescribing practices and policies post-pandemic. In January 2025, the Department of Health and Human Services proposed new rules to telemedicine prescribing of controlled substances. Under these rules, practitioners could prescribe a six-month initial supply of buprenorphine via an audio-only telemedicine interaction without a prior in-person evaluation. The DEA has yet to finalize telemedicine rules and regulations for prescribing MOUD for OUD post-pandemic, thus, studying the effects of the flexibility in telehealth buprenorphine prescribing as a result of the COVID-19 pandemic, compared to in-person care can help inform future guidelines for OUD treatment.

The goal of this study is to determine if patients receiving buprenorphine-telehealth services are more likely to be retained in treatment at six-months, compared to in-person buprenorphine services prior to the pandemic in South Carolina, a state where a quarter of the non-Hispanic Black population are living in rural areas [11]. Considering the previously observed differences in overall demographics, it is important to evaluate the impact of telemedicine-based buprenorphine prescribing and identify strategies to broaden its accessibility in this context. As an exploratory aim, we examined differences in buprenorphine retention across race, given known racial disparities in buprenorphine treatment. We hypothesized that individuals seen via telemedicine will have higher rates of retention in treatment at six-months compared to in-person buprenorphine services and that racial disparities in treatment retention will be reduced among patients receiving telemedicine, compared to in-person buprenorphine services.

## Methods

In this retrospective cohort study, we evaluated the electronic health records (EHR) of individuals treated for OUD including a buprenorphine prescription in-person or by telemedicine within a large academic medical center in the Southeastern United States between July 1, 2018, and October 31, 2021. The Medical University of South Carolina Institutional Review Board approved this study (Protocol # 00102868). Because this study involved secondary analysis of medical records, individuals were not research participants and informed consent was not required. Records were de-identified prior to investigator access through the institutional honest broker system. We defined a telemedicine appointment as a visit conducted via audio/video and in-person as a visit conducted at a clinic where the patient was physically present. Both in-person and telemedicine visits occurred throughout the entire study.

### Study design

**Fig. 1:**
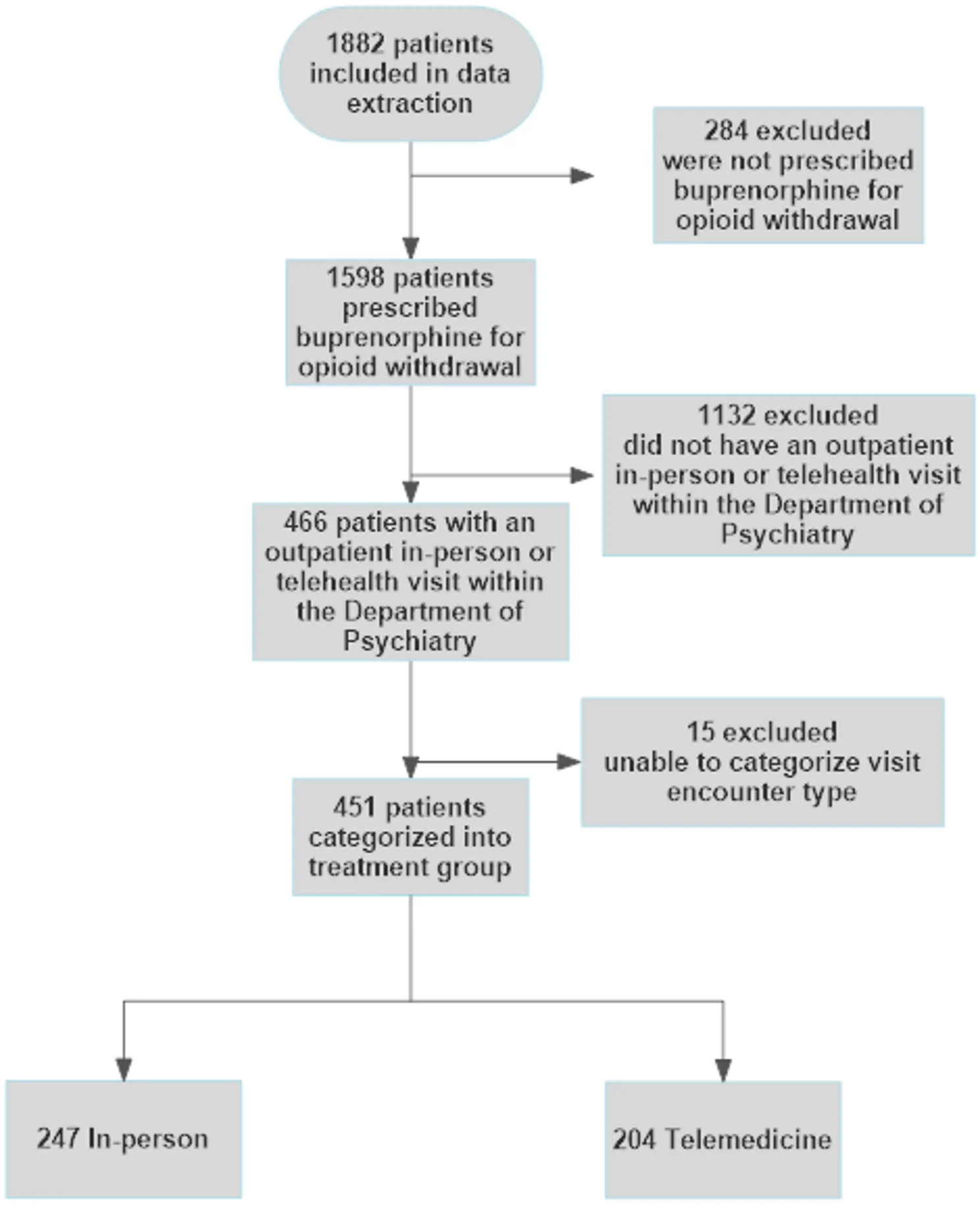
Patient inclusion and exclusion flowchart

Individuals eligible for inclusion in the study: (1) were 18 years of age or older; (2) had at least one in-person or telemedicine visit within the Department of Psychiatry at an academic medical center between July 1, 2018, and October 31, 2021; and (3) had a prescription for buprenorphine for OUD associated with an outpatient visit. Participants were excluded if they were not prescribed buprenorphine for the treatment of OUD or their visit occurred only in the hospital (vs. outpatient) setting (Fig. 1). Only outpatient visits with an associated buprenorphine prescription in the EHR were included in all analyses regardless of whether or not the prescription was filled. Data for included individuals were collected until a six-month retention time was achieved, the individual had longer than 90-days between visits, or the end of the data collection period (i.e., October 31, 2021). The primary outcome measure of this study was the percentage of individuals retained in care at six-months after their initial visit for OUD treatment within the study period. Retention in care was defined as a documented buprenorphine prescription associated with an outpatient visit within six-months of the individual’s initial visit, with no gaps in care longer than 90days over the six-month period. Routine follow-up was defined as one outpatient visit for a prescription refill for buprenorphine within 90-days of the previous visit as part of standard of care. Length of time in treatment was defined as the number of days between the initial and final visit following the criteria previously described.

### Data collection

Given the study was peri-pandemic, some patients started treatment in-person and then were transitioned to telemedicine visits. Most (87%) patients received the entirety of their treatment via one modality. Visits were categorized into two treatment groups: (1) in-person (< 50% of clinician encounters occurred by telemedicine); or (2) telemedicine (> 50% of clinician encounters occurred via telemedicine). Fifteen individuals were unable to be categorized and were excluded from further analysis. We subsequently limited our sample to 451 individuals (247 in-person and 204 telemedicine) with a total of 1850 buprenorphine order visits.

For the primary outcome analysis, individuals (*n* = 40: 37 telemedicine, 3 in-person) were excluded if their initial visit occurred after May 1, 2021, six-months before the end of the study period, to allow for assessment of the primary outcome. Data for included individuals were collected until a six-month retention time was achieved, the individual had longer than 90-days between visits, or the end of the data collection period (i.e., October 31, 2021). Age, gender, race, and insurance type at the time of the individual’s initial visit were documented. Gender, race, and insurance type were missing from one, 12, and three individuals, respectively. These individuals were excluded from the respective analyses. Race was dichotomized for data analysis due to limited variability in minoritized racial/ethnic groups. Individuals identified as American Indian or Alaskan Native (*n* = 2), Asian (*n* = 1), Black or African American (*n* = 39), or Other (*n* = 5) were categorized as non-White. All individuals with an initial visit during the study period were included in the secondary analysis evaluating length of time in treatment.

### Statistical analysis

The primary and select secondary outcomes, including the percentage of individuals retained in treatment at six-months and demographic characteristics were evaluated by Pearson chi-squared tests. We used multivariable logistic regression to calculate adjusted odds ratios (AOR) and 95% confidence intervals (CI) to determine factors associated with retention rates. Kaplan-Meier survival analysis was used to evaluate time in treatment. For individuals that remained in treatment within six-months of their index visit, time in treatment was censored. The percentage of censored cases present in each modality/race group were not similar (in-person-based/White (28%), telehealth/White (34%), in-person/non-White (52%), telehealth/non-White (46%) but were evenly distributed over time. Due to the early termination of MOUD treatment among patients, Breslow (Generalized Wilcoxon) [12] tests were used to compare the probability of remaining in treatment between groups. A Bonferroni correction was used to adjust for multiple comparisons. IBM SPSS Statistics Version 28 was used for analysis and a *P*-value of 0.05 or less was considered statistically significant.

## Results

Among the study cohort (*N* = 451), 411 had an index visit prior to May 1, 2021, and were included in the primary analysis. Individuals averaged four (range: 1–19) visits over the course of the study. Most individuals were female (64.4%), White (89.2%), and insured via Medicaid (38.7%) with an average age of 38 (range: 18–76) years (Table 1). Six-months after the index-visit, 35.3% were retained in treatment. Retention was related to visit modality with telemedicine-based individuals significantly more likely to be engaged in treatment at six-months (42.5% vs. 30.3% (*p* = 0.011) (Table 1).

**Table 1:** Patient demographics and sociodemographic factors related to treatment retention or disengagement at six-months

Covariate	Overall sample, *N* = 411		Remained in Treatment at six-months, *N* = 145 (35.3%)		*p* value
	*N*%		*N*%		
**Age**					
18–24	37	9.0%	13	35.1%	0.629
25–34	163	39.7%	61	37.4%	
35–44	101	24.6%	32	31.7%	
45–54	53	12.9%	18	34.0%	
55–64	39	9.5%	17	43.6%	
65+	18	4.4%	4	22.2%	
**Gender**					
Male	146	35.6%	52	35.6%	0.937
Female	264	64.4%	93	35.2%	
**Race**					
White	356	89.2%	118	33.1%	0.008*
Non-White	43	10.8%	23	53.5%	
**Insurance Type**					
Uninsured	93	22.8%	29	31.2%	0.117
Commercial	91	22.3%	30	33.0%	
Managed Care	25	6.1%	4	16.0%	
Medicaid	158	38.7%	65	41.1%	
Medicare	41	10.0%	15	36.6%	
**Visit Type**					
In-Person	244	59.4%	74	30.3%	0.011*
Telemedicine	167	40.6%	71	42.5%	

*Significant at the *p* = 0.05 level

Multiple logistic regression analysis indicated a significant difference in six-month treatment retention between visit type. Individuals with telemedicine visits had 1.8 times higher odds of remaining in treatment at six-months compared to those with in-person based visits (AOR = 1.87, 95% CI = 1.17–3.00) when accounting for other covariates (Table 2). Additionally, individuals insured by Medicaid had 1.9 times the odds of remaining engaged in treatment than uninsured individuals (AOR = 1.94, 95% CI = 1.05–3.58) (Table 2).

**Table 2:** Odds of remaining in treatment at six-months across sociodemographic characteristics

Covariate	In treatment at six-months	
	AOR	95% CI
**Age Class (ref. 18–24)**		
25–34	0.95	0.42–2.13
35–44	0.78	0.33–1.83
45–54	0.94	0.36–2.44
55–64	1.17	0.40–3.37
65+	0.43	0.09–2.04
**Gender (ref. Male)**		
Female	0.73	0.45–1.20
**Race (ref. White)**		
Non-White	2.05*	1.02–4.14
**Modality (ref. In-Person)**		
Telehealth	1.87*	1.17–3.00
**Insurance Status (ref. Uninsured)**		
Commercial	1.44	0.73–2.85
Managed Care	0.57	0.17–1.90
Medicaid	1.94*	1.05–3.58
Medicare	1.72	0.64–4.62

Note. Ref = reference group; AOR = adjusted odds ratios, 95% CI = 95% Confidence Interval

*Significant at the *p* = 0.05 level

**Fig. 2:**
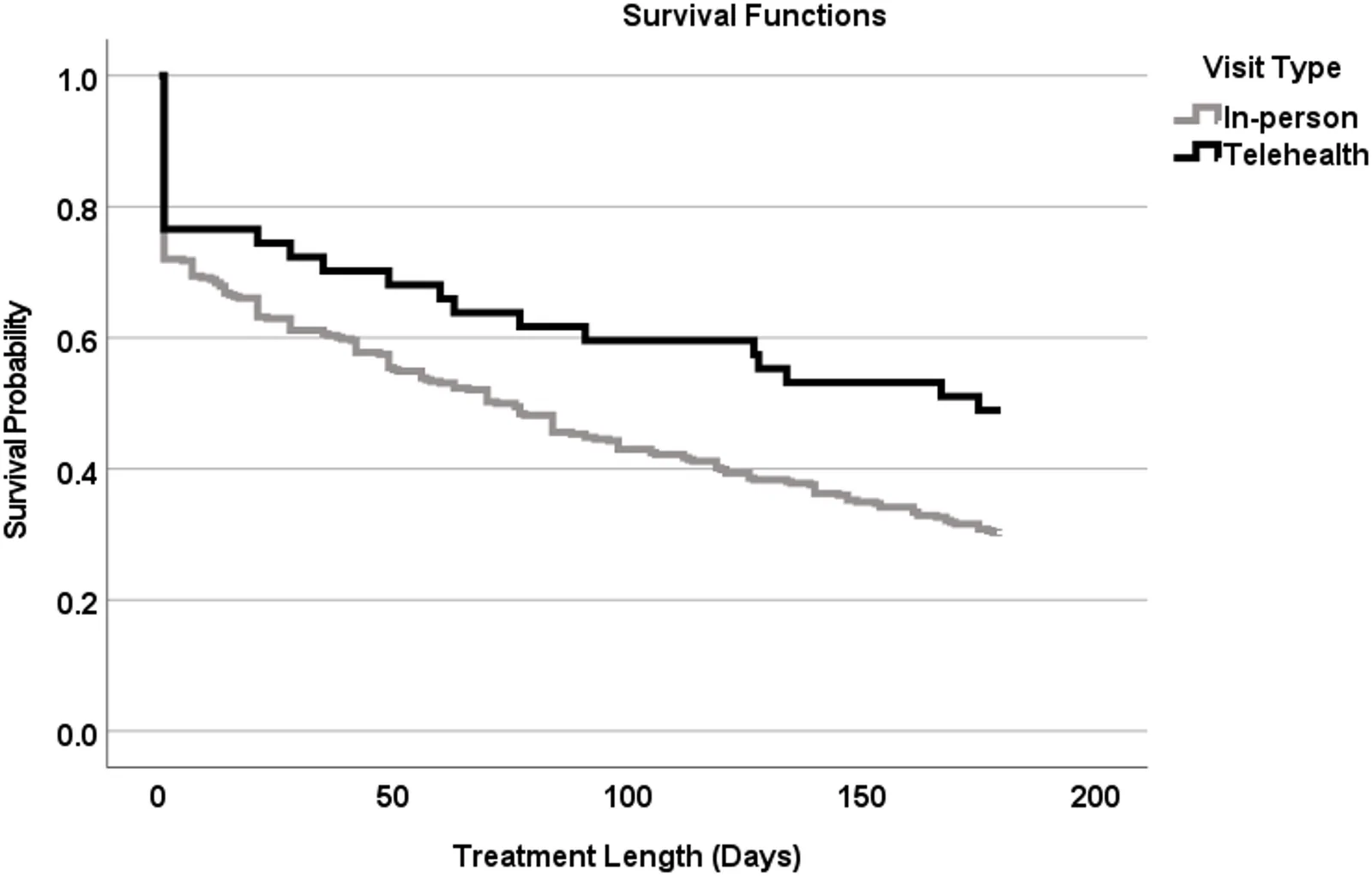
Kaplan-Meier survival analysis depicting length of time in treatment for in-person and telemedicine visit types

**Fig. 3:**
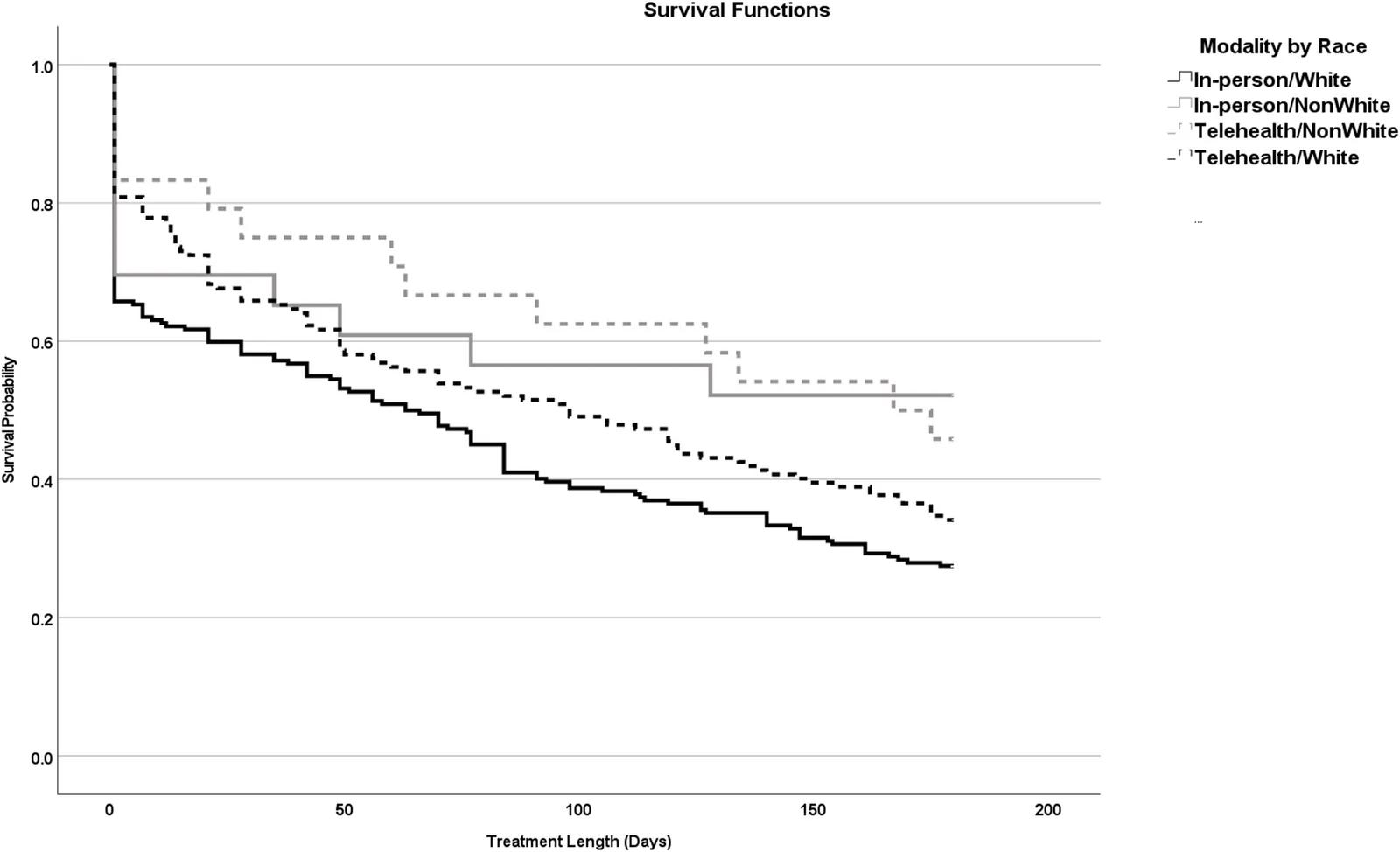
Kaplan-Meier survival analysis depicting length of time in treatment for White and non-White patients by treatment modality

Kaplan-Meier survival analysis showed that individuals engaged in telemedicine visits had a median treatment duration of 98 days (95% CI = 60–136 days) while in-person individuals had a median treatment duration of 70 days (95% CI = 53–87 days). Breslow’s test indicated the survival distribution for the two treatment types were statistically different (Χ^2^ = 4.67, *p* = 0.03) (Fig. 2; Table 3). A notable proportion of patients (27% overall) attended only their initial intake visit and did not engage in subsequent treatment, which is reflected in the Kaplan–Meier curves as discontinuation at time zero (Fig. 3). Early disengagement varied across subgroups: 34% for in-person, 20% for telehealth, 34% for in-person White, 30% for in-person non-White, 19% for telehealth White, and 17% for telehealth non-White.

**Table 3:** Mean and median number of days to treatment discontinuation based on treatment modality and race. Median number of days to treatment discontinuation represents the time at which point half of the patients disengaged from treatment

	*N*	Mean Time (Days) to Treatment Discontinuation	SE	95% CI	Median Time (Days) to Treatment Discontinuation	SE	95% CI	*p*-value
**Visit Type**								
Office-based	247	83	4.89	73–93	70	8.81	53–87	0.03*
Telemedicine	204	97	5.33	86–107	98	19.56	60–136	
**Race**								
White	389	87	3.87	79–94	76	10.11	56–96	0.04*
Non-White	47	113	11.30	91–135	175			
Overall	451	90	3.68	82–97	77	9.49	58–96	

### Racial differences in treatment retention (exploratory analysis)

Non-White individuals were significantly more likely to remain engaged in treatment at six-months than White individuals (66.9% vs. 46.5%, *p* = 0.008) (Table 1). Multiple logistic regression analysis indicated that non-White individuals had twice the odds of remaining in treatment at six-months as compared to White individuals (AOR = 2.05, 95% CI = 1.02–4.14) (Table 2,). Kaplan-Meier analysis showed that White individuals had a median treatment duration of 76 days (95% CI = 56–96 days) while the median was not reached for non-White individuals (i.e. 50% of patients did not leave treatment within 180 days). Breslow’s test indicated a difference in survival distributions (Χ^2^ = 4.15, *p* = 0.04) (Table 3).

When adjusting for race, there was a significant difference in treatment length between telemedicine and in-person visits (*p* = 0.018). Non-White individuals with telemedicine visits remained in treatment the longest at an average of 119 days, 12 days longer than non-White in-person individuals (mean = 107 days) and 23 days longer than White individuals with telemedicine visits (mean = 96 days) (Table 4; Fig. 3). Breslow’s (Generalized Wilcoxon) pairwise comparisons showed no significant difference between modality and racial groups after applying a Bonferroni correction, likely due to small sample size.

**Table 4:** Average number of days to treatment discontinuation across race and visit type

Visit Type	White		Non-White	
	*N*	Mean Time (Days) to Treatment Discontinuation M (SD)	*N*	Mean Time (Days) to Treatment Discontinuation M (SD)
In-Person	222	80 (70–90)	23	107 (74–140)
Telemedicine	167	96 (84–107)	24	119 (90–148)
Overall	389	87 (79–94)	47	113 (91–135)

Note. M = Mean; SD = Standard Deviation

## Discussion

As a result of the COVID-19 pandemic, regulations for prescribing buprenorphine via telemedicine were temporarily removed to facilitate access to this life-saving treatment. Consequently, MOUD became more widely accessible and there was improved retention in treatment compared to pre-pandemic, in-person MOUD services [8]. This study is consistent with the existing literature that those receiving buprenorphine via telemedicine are more likely to be retained in treatment compared to those receiving buprenorphine via in-person care at six-months.

While racial disparities in accessing in-office buprenorphine are well-established [3, 13], little is known about whether telemedicine can reduce racial disparities and improve retention in treatment. Contrary to two prior studies, our results suggest that non-White patients are twice as likely to be retained in buprenorphine telemedicine treatment at six-months, compared to White patients [9, 10]. Results suggest that telemedicine is associated with improved retention in treatment among racially minoritized patients receiving buprenorphine and may represent an important strategy to achieve health equity in the treatment of OUD. Additional research is necessary to examine retention outcomes across different racial groups and treatment modalities, given the small sample of American Indian or Alaskan Native and Asian participants in the current study and the study’s conflicting results with two prior studies.

In prior studies, preference for telemedicine primary care visits varies by age and race, with Black patients more likely to choose both video and telephone visits compared to White counterparts [14]. Some potential reasons why non-White individuals in our sample were retained in OUD treatment longer via telemedicine may be that the preference for telemedicine in primary care may also generalize to substance treatment. In addition, substance use stigma is a deterrent for seeking treatment particularly for marginalized populations, such as racial and ethnic groups, who face a “double stigma” [15]. Telemedicine can offset this perception as it limits interactions with ancillary staff, allows access to treatment in the comfort of one’s own home, and improves perceived privacy [16, 17]. Our study results may differ from those in Appalachia and Northern States potentially due to cultural differences in this Southern state, leading to discrepancies compared to non-White populations in other regions [18]. However, a common limitation across the telehealth literature is the small number of non-White participants, underscoring the need for larger, geographically diverse studies to more fully examine these differences.

Buprenorphine treatment for OUD is associated with a myriad of beneficial clinical outcomes including reduced rates of illicit opioid use, treatment dropout, healthcare utilization, and mortality [19–21]. Patients that receive long-term MOUD treatment are 50% less likely to die due to overdose, compared to those not receiving MOUD [2] yet, access to MOUD, and buprenorphine specifically, is disparate. Buprenorphine is disproportionally underutilized among racial/ethnic minorities [22, 23] and is more accessible to higher income and predominantly White communities [24–26]. Racially and ethnically minoritized individuals are more likely to be prescribed methadone [25–27]. Across all racial/ethnic groups, retention in MOUD treatment is poor and variable across settings (average retention rates are 30–50%), with marginally higher retention in methadone treatment relative to buprenorphine treatment [28–30]. Consistent with prior studies, the steep initial drop in the Kaplan-Meier survival curve (Fig. 3) reflects early disengagement with more than one-quarter of the sample only attending their initial visit. Notably, when minoritized groups are prescribed MOUD, they are retained in (in-person) treatment as long or longer than their White counterparts [22, 31]. Additionally, all patients in the study receiving MOUD treatment via telemedicine were retained in treatment longer than patients who were engaged in in-person services prior to the COVID-19 pandemic. Utilizing telemedicine for the medical management of OUD represents one potentially critical strategy to improve retention in MOUD, and in reducing racial disparities in treatment retention.

Our study demonstrates that those insured by Medicaid are more likely to receive treatment via telemedicine compared to those with other types of insurance or uninsured. These findings are not surprising given that Medicaid is the largest payer for OUD treatment in the US and prior work has demonstrated that Medicaid enrollees with OUD are more likely to receive MOUD treatment compared to commercial enrollees [32, 33]. (The difference in MOUD treatment by insurance type is rather small (~ 9% higher) and remains unexplained even after controlling for patient and provider characteristics [33]. (. Our findings demonstrate a larger difference in treatment rates by insurance type and telemedicine suggesting that having both Medicaid and telemedicine increases the accessibility of MOUD treatment.

There are numerous barriers to MOUD receipt which can be amplified for racially and ethnically minoritized patients. Stigma towards people who use opioids and MOUD is a significant barrier to OUD treatment entry and retention [34]. Stigma towards individuals receiving MOUD is held by some recovery communities, healthcare providers, and even patients (internalized stigma) who erroneously equate MOUD as a “substitution” for illicit opioids [35]. Fear of judgment based on previous experiences with stigma in the healthcare setting deter people from seeking MOUD [36]. Additionally, stigma from the general public is a driver of limited access to evidence-based treatment including MOUD, as it perpetuates misinformation, stereotypes, prejudice, and discrimination towards people who use opioids, serving as a significant treatment deterrent [37]. Additional barriers include MOUD treatment “deserts” and lack of available providers, and structural barriers such as lack of time, limited or no transportation, and cost [38], all of which disproportionately affect communities of color and other underserved populations and further exacerbate racial disparities [39, 40]. Telemedicine can improve trust, comfort, and stigma [16, 17], while also overcoming the need for proximity to providers and barriers such as limited transportation [41]. 

## Limitations

There were several limitations to the study. First, the study design was a single site, retrospective study, relying on EHR data to represent in-person and telemedicine buprenorphine services. While visits in the EHR are coded using a telemedicine specifier for a telehealth appointment, scheduling errors may have occurred. Future studies could seek to replicate these findings using multiple sites randomized controlled trials. Relatedly, we only reviewed EHR data from a predetermined timeframe and identified the first OUD-related visit within that period (i.e., July 1, 2018 to October 31, 2021) as the initial visit. It is possible that we inadvertently underestimate patients’ time in treatment if their first OUD-related visit took place prior to July 1, 2018. A second pandemic-related limitation is possible changes to prescribing practices, as some clinics moved to a more flexible prescribing approach during the height of the pandemic to accommodate limited contact mandates (e.g., quarantine, social distancing protocols). Third, the sample sizes between the white and Non-white populations are notably unbalanced (*n* = 356 vs. 43) and could contribute to the larger standard deviations observed for days to treatment discontinuation. This imbalance may influence the observed differences, including the potential for reduced statistical stability and generalizable for the Non-white subgroup. Although our use of non-parametric tests helps mitigate concerns related to unequal variance and the results remain consistent across analytic approaches, the results for this subgroup should be interpreted with caution and highlight the need for larger, future studies with more diverse and adequately powered samples. Fourth, patients’ racial and ethnic identities were dichotomized due to limited variability in racial/ethnic groups, preventing a more nuanced understanding of whether telemedicine equally improved buprenorphine retention across all minoritized racial and ethnic individuals or only certain groups. This limitation represents an important area for future research. Lastly, we were unable to determine which illicit opioid(s) patients in this study were using or had used prior to treatment. Differences are seen across individuals using various opioid types in regard to sociodemographic characteristics (e.g., income, age), co-occurring conditions (e.g., depression), and treatment outcomes (e.g., buprenorphine induction and retention) [42–44]. It is possible that individuals using different types of opioids may respond differently to telemedicine treatment, though this remains an empirical question. Study strengths include a large sample of “real world” clinic data representing individuals in the community living with OUD who were seeking treatment, and longitudinal data across a six-month period.

## Conclusion

With the removal of the “waiver” requirement to prescribe buprenorphine, access to this life-saving medication has greatly improved, especially for some of the most vulnerable and at-risk patients experiencing healthcare disparities. However, access to MOUD is still limited and barriers persist. Telemedicine is one solution to overcome barriers to buprenorphine treatment. Our results suggest that using telemedicine to prescribe and manage buprenorphine for OUD enhances retention in treatment compared to in-person buprenorphine management, with notably improved retention for racial minorities. Telemedicine overcomes structural and societal barriers to attending appointments which disproportionately impact minority groups and thus potentially facilitate greater retention in care. These findings, together with a larger body of observational work suggesting telemedicine-based services enhances MOUD access and retention [6, 7], advocate for removal of restrictive buprenorphine prescribing regulations (i.e., required in-person visits), which would facilitate more effective and sustained access to life-saving treatment especially during the escalating opioid overdose crisis. As the guidelines and regulations for telemedicine are being determined, this study contributes to the growing body of evidence that shows an improvement in buprenorphine treatment retention with telemedicine utilization.

## Data Availability

The datasets used and/or analyzed during this study are available from the corresponding author on reasonable request.
